# Involving and engaging pregnant women in maternity-related research: reflections on an innovative approach

**DOI:** 10.1186/s40900-021-00332-8

**Published:** 2021-12-16

**Authors:** Laura Goodwin, Magdalena Skrybant, Sara Kenyon

**Affiliations:** 1 School of Health and Social Wellbeing, University of the West of England, Glenside Campus, Bristol, BS16 1DD UK; 2 Institute of Applied Health Research, Murray Learning Centre, University of Birmingham, Birmingham, B15 2TT UK

**Keywords:** Patient and public involvement, Research engagement, Pregnancy, Pregnancy yoga, Exercise in pregnancy, Maternity research, Model of engagement, Ethnic inequality

## Abstract

**Background:**

Meaningful public involvement in maternity research remains challenging, partly due to the transient nature of pregnancy. This paper reflects on the development, implementation and simple evaluation of an innovative and inclusive approach to engaging and involving pregnant and early postnatal women in research.

**Methods:**

Between January and February 2018, a Research Fellow in Maternity Care, a Professor of Evidence Based Maternity Care, and a Patient and Public Involvement Lead convened for a number of meetings to discuss how public involvement and engagement might be improved for pregnancy-related research. A stakeholder group was created, including a local community matron, a community engagement officer at a local children’s centre, public contributors, and senior members of the Maternal and Child Health theme of the West Midlands Collaboration for Leadership in Applied Health Research and Care (CLAHRC WM). The team worked together to develop a format for Yoga for Bump sessions: a free 90-min session, offered weekly, which included research involvement/engagement, pregnancy yoga, and a ‘question and answer’ session with a midwife.

**Results:**

A total of 67 women from two local communities in Birmingham attended Yoga for Bump sessions, which ran between May and December of 2018. Evaluation of the sessions suggested benefits to both women and researchers: it created mutually beneficial relationships between contributors and researchers, provided opportunities for women to engage and get involved in research that was directly relevant to them, and provided a convenient and efficient way for researchers to involve and engage pregnant women from diverse backgrounds in their research. Unintended benefits included self-reported improvements in women’s health and wellbeing.

**Conclusions:**

Yoga for Bump demonstrates an innovative approach to engaging and involving pregnant and early postnatal women; combining a free exercise class with healthcare advice and opportunities to engage with and be involved in research, and demonstrating mutual benefits for those involved. This model has the potential to be replicated elsewhere to support inclusive public involvement in pregnancy-related research. Further work is needed to design and evaluate similar approaches to involvement/engagement and explore potential funding avenues to enhance sustainability.

## Plain English summary

Making sure that the public are involved in research is really important. It can sometimes be hard for pregnant women to get involved with research because they are only pregnant for a short amount of time and they are often busy with other things. This means that the research might only feel directly relevant and important to them for a short time. We designed a new way to encourage pregnant women to get involved and engaged in research. We did this by offering a free pregnancy yoga class to women. This class included a ‘question and answer’ session with a midwife and a discussion with a researcher about some research to do with pregnancy. We ran two classes a week, in two different parts of Birmingham, United Kingdom (UK). The classes took place between May and December 2018 and 67 different women attended the classes. We wanted to see if this was a good way to involve and engage pregnant women in research, so we did a simple evaluation. We used some questionnaires and notes that we had made. We found that the sessions were helpful for both women and researchers. Women enjoyed being involved in the research and told us they had felt healthier and less stressed from the yoga. Researchers found it really useful to be able to talk to women from lots of different backgrounds and experiences. There were some difficult parts of running the sessions, like the costs, and the time needed from us to make sure sessions ran smoothly.

## Background

There is a growing evidence base which supports the value of involving patients and the public in health research. In addition to ensuring the relevance and suitability of research, meaningful public involvement can improve the quality of the research outcomes [1–3]. Further benefits of meaningful public involvement throughout the research cycle include ensuring that the research methods used are acceptable from practical and ethical perspectives [4, 5], and facilitating dissemination of research findings in ways that are accessible by, and appropriate for, audiences relevant to the focus of the study.

The National Institute for Health Research (NIHR), a key funder for health and social care research in the United Kingdom (UK), defines public involvement as research done ‘with’ or ‘by’ the public, not ‘to’ ‘about’ or ‘for’ them [6]. A global drive towards more embedded involvement has become increasingly important in UK healthcare research, as reflected by the directive from ‘Liberating the NHS’: “no decision about me without me” [7]. Ensuring that public contributors are embedded in a meaningful way throughout the research cycle is an expectation for UK health and social care research, and in November 2019 the NIHR launched UK Standards for Involvement to improve the quality and consistency of public involvement in health and social care research [8].

Despite these Standards, and the clear benefits of embedded involvement, it can be challenging to involve patients and the public in ways that are meaningful and that have impact. Involving pregnant contributors in research has proven especially challenging, as issues relating to maternity care may only feel directly relevant to contributors for a limited amount of time [9]. Consequently, there is a need for a constant ‘throughput’ of women with recent experiences of accessing maternity services who can provide insights into current service provision, complementing perspectives from longer-standing public contributors. This contrasts with other areas of health research where a ‘standing group’ of contributors (e.g. people with long-term conditions who have regular access to services and continuing/recent experiences) continue to benefit from involvement/engagement in research relevant to them. The weeks and months following birth can also be an especially challenging time for research involvement and engagement, due to competing demands on time associated with caring for an infant, return to work, and the decreasing relevance of maternity services.

It is well-recognised that the most disadvantaged or marginalised members of the public are under-represented in research [10, 11], however it can also be challenging to include these individuals in research engagement and involvement activities. In the UK, ethnic minority groups are especially under-represented in medical research [12] as well as patient and public involvement activities [13]. In 2015, a strategic review of public involvement in England highlighted the need for public involvement to become more diverse and inclusive, and noted barriers for researchers and public contributors when trying to work with different communities and populations [14]. However, the most recent survey conducted by NIHR on public involvement suggests that young people and minority ethnic communities remain under-represented in public involvement; only 16% of NIHR public contributors surveyed were under 50 years old, Asian ethnic groups represented only 3% of respondents, and Black ethnic groups only 2% [15]. For maternity and postnatal research, this disparity is of particular concern. UK reports on maternal and perinatal mortality suggest that women from minority ethnic backgrounds are significantly more likely to lose their babies or die during or after their pregnancy compared to their White British counterparts [16, 17]. Following these reports, a letter published in the British Medical Journal called for ‘urgent action’ to tackle the ‘systemic biases contributing to unequal mortality outcomes in ethnic minority women and women facing multiple problems and deprivation’ [18]. Increased involvement in research from diverse communities is part of addressing this issue.

Like other areas of research, there are traditional barriers to involvement in maternity-related projects, such as lack of time to become involved in research, transport issues, social contexts, communication issues and caring commitments [10, 13]. Current strategies to recruit public contributors may not attract typical members of the target population(s) [19], and constraints on time and funding to proactively recruit appropriate contributors can act as barriers to involvement in the crucial stages of research development [19–22]. It is therefore necessary for research teams to vary their approaches to involvement [23], and pay greater attention to ways in which they might work more flexibly with local communities to create opportunities for involvement [24], especially those from minority ethnic communities.

With funding from the Wellcome Trust Public Engagement Fund (Ref: 210559/Z/18/Z), a team of researchers and a Public Involvement Lead developed and piloted an innovative and inclusive approach to engaging and involving pregnant and early postnatal women in research. This took the form of a free pregnancy yoga class, delivered in community settings, with integrated opportunities to involve and engage women in research: Yoga for Bump. This paper reflects on the development, implementation and simple evaluation of this novel approach.

## Methods

### Aims

**Fig. 1:**
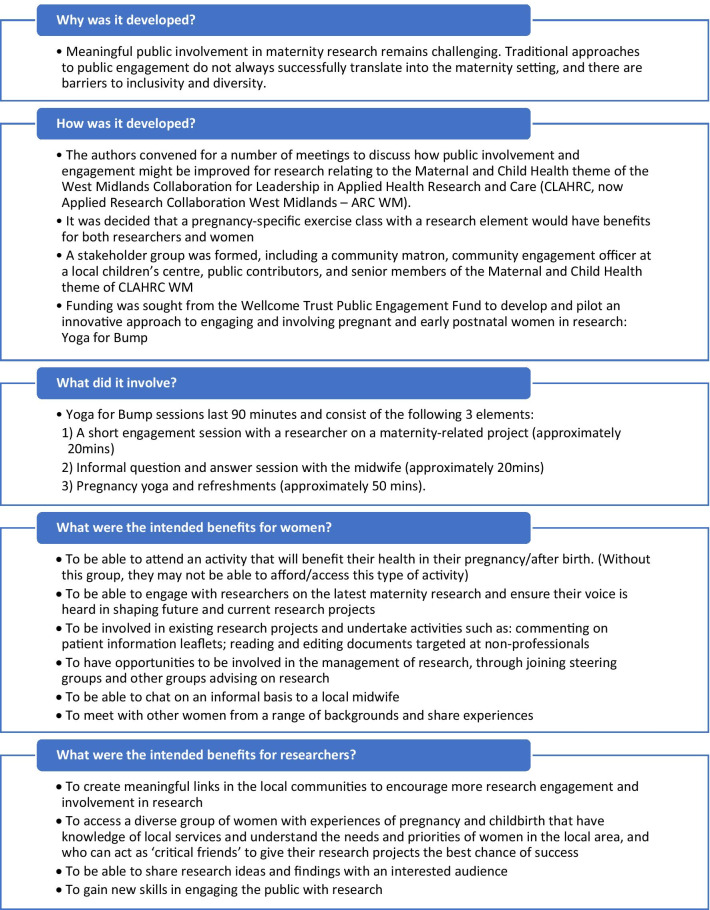
Summary of the aims and design of the Yoga for Bump project

The key aim of this project was to explore an alternative approach to engaging and involving pregnant women in research that moved beyond traditional methods of involvement, and which offered benefits to women over and above that resulting from their involvement/engagement in research. Typically, involvement is organised in settings convenient to researchers (e.g. University campus or hospital location) and during the working day. Meetings for discussions with public contributors may last several hours to maximise time for discussion and mechanisms for offering honoraria to contributors are notoriously complex. This innovative approach to involving and engaging pregnant women was centred around developing links with women in two local communities through mutually beneficial activity. Spaces to engage and involve women were in local community settings and linked to a focussed activity, pregnancy yoga. More detailed aims of this design can be found in Fig. 1.

### Design of the sessions

Between January to February 2018, the authors (a Research Fellow in Maternity Care, a Professor of Evidence Based Maternity Care, and a Patient and Public Involvement Lead) convened a number of meetings to discuss more inclusive approaches to public involvement and engagement in the Maternal and Child Health theme of the West Midlands Collaboration for Leadership in Applied Health Research and Care (CLAHRC WM, now Applied Research Collaboration West Midlands – ARC WM). While involvement activities for the theme had previously included accessing existing groups at local children’s centres, several limitations of this approach were noted. For example, access to existing groups had proven difficult to organise, women did not always meet the intended demographic (i.e. pregnant or early postnatal) and the number of women in attendance at these groups often varied. It was agreed that a new approach to involvement would be beneficial, and should focus on inclusivity and mutual benefits to women and researchers. The lead author had previous experience of successfully engaging women in research activities during a university-organised pregnancy-specific exercise class. It was therefore proposed that a specialist pregnancy exercise class might be developed, which would incorporate a research involvement/engagement element.

After searching the literature on the benefits of different exercise in pregnancy, discussions on these findings were held with the maternity theme public contributors and researchers. From these discussions, it was decided that a pregnancy yoga class might be the most suitable approach. Yoga is a low-impact, low to moderate intensity exercise, which may effectively decrease stress levels, anxiety scores, depression scores, and pain response as well as increasing maternal immunity and emotional-wellbeing [25]. There is also evidence to suggest that exercise including mind–body practices, such as yoga, may be particularly effective in addressing both the physical and emotional aspects of pregnancy and labour [26]. Further consultation with the theme public contributors and a search of local antenatal exercise classes suggested that yoga was the most popular choice amongst pregnant women, alongside ‘Aquanatal’ sessions.

The lead author became aware of a pregnancy yoga teacher who was also a midwife at a local National Health Service (NHS) hospital Trust, who agreed to be involved in creating a specialised class. A stakeholder group was created, including a local community matron, a community engagement officer at a local children’s centre, public contributors, and senior members of the Maternal and Child Health theme of CLAHRC WM. The team worked together to develop a format for Yoga for Bump sessions, which would benefit both women and researchers. The group discussed potential barriers to involvement and made several key decisions about the sessions. Firstly, there would be no costs for attending the sessions: it was considered important to remove any potential financial barriers. Secondly, it was agreed that women would not need to ‘sign up’—the sessions would be advertised in local communities and places offered on a ‘first come, first served’ basis. It was agreed that Yoga for Bump sessions should last 90 min and consist of the following three elements:

1. An engagement/involvement session with a researcher on a maternity-related project (approximately 20 min)
2. Informal question and answer session with the midwife (approximately 20 min)
3. Pregnancy yoga and refreshments (approximately 50 min).

Women were not obligated to attend all parts of the session, and could choose to attend any or all parts. Given challenges of time to explore topics with pregnant women, researchers were able to return for more than one session, and could offer women further opportunities for research involvement outside of the Yoga for Bump setting. More detail on this design can be found in Fig. 1.

### What we did

**Fig. 2:**
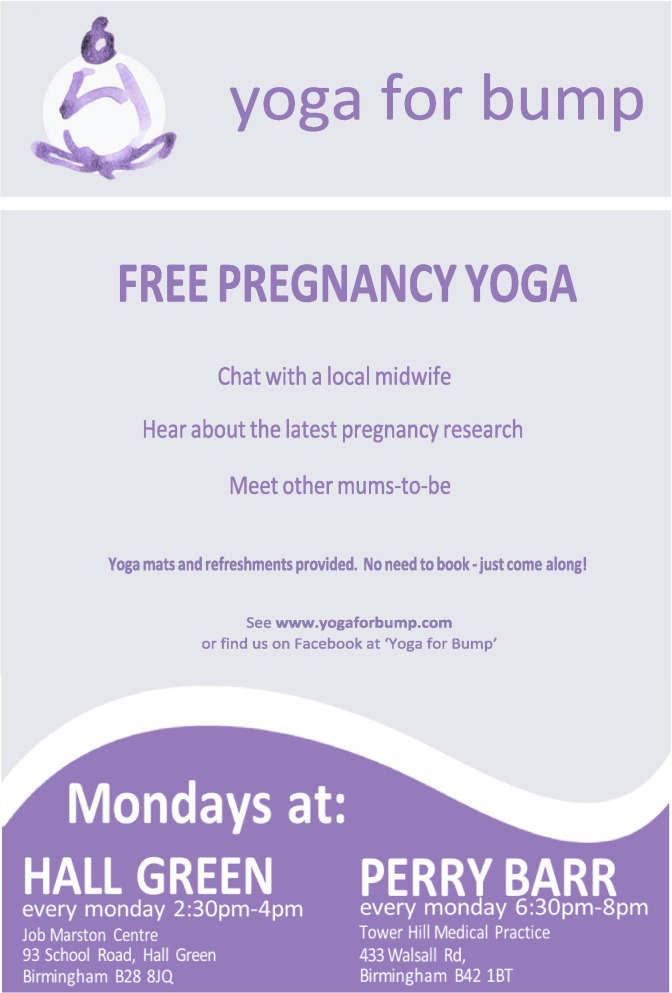
Advertisement poster/leaflet for Yoga for Bump sessions

**Fig. 3:**
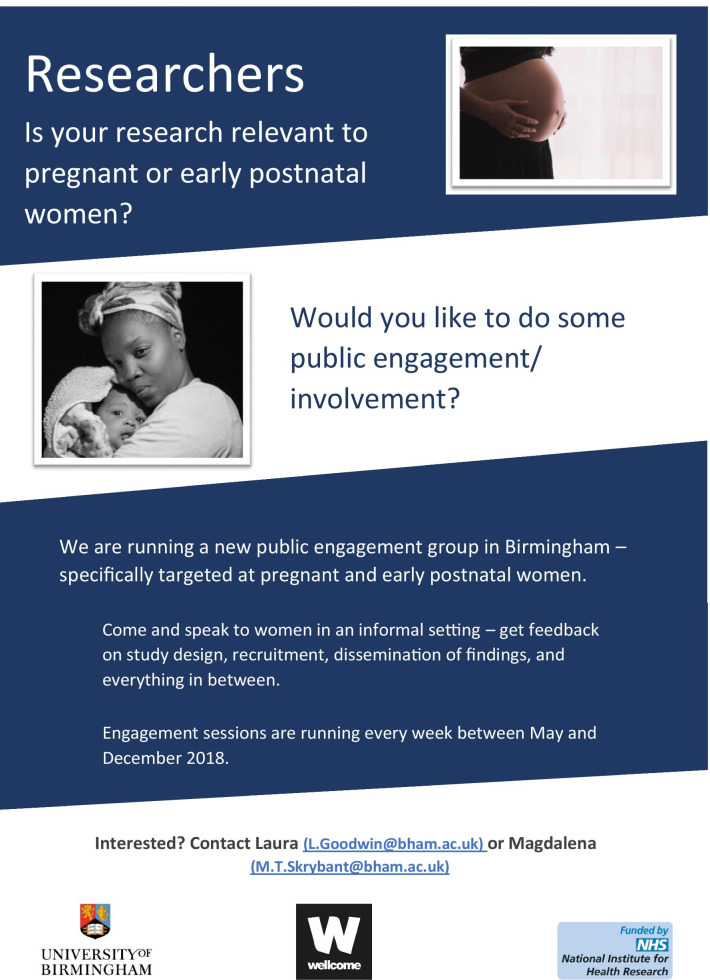
Poster advertising Yoga for Bump sessions to researchers

Two separate Yoga for Bump sessions were offered weekly in Birmingham, UK. So as to include women from diverse social demographics, the two sessions were held in separate locations and at different times. Consultations were held with our stakeholder group to identify suitable locations for classes, which would allow us to serve women from diverse social and economic backgrounds. One class took place in Hall Green (postcode—B28) on a weekday afternoon (2:30-4 pm), while the other class took place in Perry Barr (postcode—B20) on a weekday evening (6:30-8 pm). Using the English Index of Multiple Deprivation (IMD) 2015, Hall Green was seen to have a lower deprivation rank, and a higher rank for income, employment and education than Perry Barr [27]. Sessions were advertised to women using leaflets and posters (Fig. 2), which were handed out by local midwives and community centre managers, and displayed in local community centres, children’s centres, General Practice (GP) waiting rooms, and on social media (including Yoga for Bump Twitter and Facebook accounts). Sessions were advertised to researchers using a poster (Fig. 3) which was displayed in University staff rooms, and emailed to local NHS Trusts and Universities. A blog post about the project aims was also published in the local CLAHRC WM newsletter.

The majority of sessions were facilitated by one or two of the project team (LG and MS), who would introduce guest researchers and facilitate group discussions. Some sessions involved research engagement (i.e. listening to a summary of research findings, and providing ideas for dissemination), whilst others were structured to include research involvement (i.e. discussing potential research questions/methodologies). All guest researchers were required to complete an expression of interest form including a summary of their project, any potential ethical issues or sensitive content, motivations for engagement, potential benefits to the women involved, and any materials they would be presenting. This was reviewed by the research team for suitability and legitimacy before the guest researcher was given an available date/timeslot to attend a session. Some researchers booked two or more slots, while some only attended one session.

All resource costs (excluding study team facilitation) were covered by the Wellcome Trust Public Engagement Fund award (Ref: 210,559/Z/18/Z), including equipment (yoga mats), venue hire, marketing, staff (yoga teacher) and refreshments.

### Evaluation

The study team prepared a protocol for a simple evaluation of Yoga for Bump, which was reviewed by the stakeholder group, including public contributors from ARC WM. The overall aim of this evaluation was to determine the success of the research engagement activities, through exploring:

1. The practical consideration of the sessions (including barriers and facilitators)
2. The perspectives and experiences of researchers and women
3. Level of research engagement and/or involvement following sessions.

This involved analysis of the following data: (1) routinely collected data; (2) questionnaires; (3) fieldnotes and reflexive accounts.

### Routinely collected data

The number of women attending each Yoga for Bump session was recorded by a member of the study team and entered on to a Microsoft Excel spreadsheet. Data were analysed using simple descriptive statistics.

### Questionnaires

To investigate researchers’ and women’s views and experiences of the Yoga for Bump sessions, a simple questionnaire was administered. Questions were set by the Wellcome Trust, as part of the process of reporting to the funder, and a few additional questions were created by the study team and reviewed by members of the stakeholder team, which included a public contributor.

All researchers who attended a research engagement session between May and December 2018 were contacted via email by one of the study team, provided with a participant information sheet, and invited to complete a questionnaire. Participants were offered the option of paper or electronic return, and consent was assumed if participants completed and returned the questionnaire. Questions focused on the researcher’s experience of attending the group, presented as a 5-point Likert scale. A mixture of multiple choice and free-text questions was included to explore why the researcher attended the session(s), what they hoped to get out of it and whether or not they were attempting to engage women outside of the sessions. If researchers did involve/engage with women outside of the session, questions explored what form these activities took. Importantly, questions explored whether researchers changed any aspect of their research as a result of attending the Yoga for Bump sessions.

Women were eligible to take part in the evaluation if they had attended one or more Yoga for Bump session and were able to understand and consent to participation. All women who attended sessions during the final month of the project (December 2018) were given a participant information sheet and invited to complete a questionnaire, either as a paper copy or via an online link. This was done by a member of the ARC WM team who the women had not met, to avoid bias in the results. This external team member gave a brief explanation of the evaluation and explained that all questionnaire data would be anonymous. Consent was assumed if participants completed and returned the questionnaire. Questions focused on women’s views and experiences of the sessions, presented as a 5-point Likert scale. A mixture of multiple choice and free-text questions was included to explore their motivation to attend the sessions, their level of engagement with the research presented, and their intention to get involved in future research. Basic demographic information (including age, ethnicity, and parity) was also collected.

Paper copies of questionnaire responses were collated by the study team and entered onto a bespoke Microsoft Excel spreadsheet. Online questionnaire data exported from secure online servers and entered onto the same database. Quantitative questionnaire data were analysed using simple descriptive statistics. Qualitative data from free-text responses were analysed thematically and managed using the Framework Method [28]. The first step was familiarisation with the data, where the lead researcher (LG) typed the handwritten free-text comments into a Microsoft Excel spreadsheet, and read each comment several times. Responses were then ‘coded’: passages of text were given a paraphrase or label that described the main idea described by the text. These codes were used to build a framework matrix in a further sheet of Microsoft Excel. Rows were used for individual participants, columns were used for each individual code, and cells were populated with data. Due to the small amount of data collected, all responses were coded in the process of creating the framework, and no further iterations were needed.

### Fieldnotes and reflexive accounts

To evaluate the success of setting up and maintaining the Yoga for Bump sessions, the project leads (LG and MS) recorded regular fieldnotes and reflexive accounts. Data were imported into the data management software NVivo 10, and analysed thematically by the lead researcher (LG) [28].

### Ethical consideration

Full ethical approval was received from the University of Birmingham Science, Technology, Engineering and Mathematics Ethical Review Committee (ERN_18-0967). Participants received written and oral information about the evaluation, and informed consent was assumed if a participant completed and returned a questionnaire.

### Patient and public involvement

Public contributors linked to ARC WM’s Maternity Theme were involved in the conception, design and evaluation of the Yoga for Bump sessions. This included helping to design the advertisement materials and strategy, the evaluation protocol and evaluation materials. A public contributor was part of the stakeholder group.

## Results

A total of 67 individual women from the two local communities attended Yoga for Bump sessions, which ran between May and December of 2018. During this time period, there were a total of 371 attendances across the 60 classes provided: 31 classes at Hall Green, and 29 classes at Perry Barr. Class size at Hall Green (2:30–4 pm) ranged from 1 to 6 with a median attendance of 4 women per class. At Perry Barr (6:30–8 pm), class size ranged from 2 to 15 with a median of 9 women per class.

### Questionnaires

#### Researcher’s experiences

Questionnaires were returned by all seven researchers who had attended Yoga for Bump sessions. Five had used the sessions once, and two had attended sessions more than once. All participants (7/7) indicated that the sessions met their needs and the resulting engagement had added value to their research. The majority (5/7; 71%) of researchers reported changing some aspect of their research as a result of their engagement sessions with women.

The majority (5/7; 71%) of researchers suggested that Yoga for Bump sessions were “extremely helpful” for engaging pregnant and early postnatal women in their research, one reported the sessions as “largely useful” and one reported the sessions as “moderately useful”. When asked about the women attending the sessions, the majority of researchers felt that they were an appropriate audience for their research; 3/7 (43%) researchers felt that the women were an “extremely appropriate” audience, 3/7 (43%) felt they were “largely appropriate” and 1/7 (14%) felt they were “moderately appropriate”.

Most (5/7; 71%) researchers who attended a Yoga for Bump session felt that they, or their employer, might consider paying to attend this type of engagement session in future.

Free-text responses suggested that researchers valued not having to organise sessions themselves, and enjoyed meeting a variety of women with differing views.

A range of women with different experiences and backgrounds participated in the discussion. It was very helpful to have an experienced patient and public involvement facilitator and a midwife who knew women from before to help engage women during the session. We collected rich information helpful for other aspects of the project beyond the main question we wanted to get input on. (R3)

I got the information I needed, and it was really easy as a researcher to be able to slot into a group without having to identify women. (R6)

#### Women’s experiences

Questionnaires were returned by all 20 women who received them. Participant demographics are reported in Table 1, and are representative of the larger group attending sessions.

**Table 1:** Demographic characteristics of women completing an evaluation of Yoga for Bump sessions

Demographic variables	No. (%)
Age range	
26–30	4 (20)
31–35	11 (55)
36–40	5 (25)
Ethnicity	
White	10 (50)
Black	4 (20)
Asian	1 (5)
Mixed	4 (20)
Other	1 (5)
Pregnancy	
First pregnancy	11 (55)
Second or more	9 (45)
Maternity care providers	
Midwives	11 (55)
Midwives and consultants	9 (45)

The completeness of questionnaires varied, and findings below are reported as a percentage of the number of women answering each individual question.

All (20/20) participants said that they would recommend Yoga for Bump sessions to friends to friends and family. Almost all (16/17; 94%) said that they had learnt something new from the research part of the sessions, and 15/16 (94%) felt that it was important that this research engagement was taking place. The majority (9/15; 60%) indicated that they found the research presented at the sessions “extremely interesting” or “largely interesting", and the remainder (6/15; 40%) selected “moderately interesting”. Most (12/16;75%) participants said that they would be “somewhat likely” or “very likely” to get involved in research in the future, with the other 4 (25%) reported being “unsure”.

Free-text responses from women suggested that they had experienced unexpected benefits from attendance from the classes, for example making new friends in the area, sharing pregnancy concerns with a group of other pregnant women, and feeling able to talk about 'taboo' subjects with confidence.

[I’ve enjoyed] the communal atmosphere and friendliness of the group, and being able to talk about miscarriages without upset. (P4)

Women also reported that they perceived the yoga element of the sessions had improved their health and wellbeing, and that attending these sessions had helped them with managing day-to-day stress, and feeling better prepared for labour and childbirth.

I liked being able to ask questions to the midwife, learning how to relax in labour through breathing (something I've applied in life in general), and the social aspect of the class. (P14)

The only negative comments referred to the fact that the project had an end date, and that the sessions could have been longer.

I’m sad that the sessions are ending soon. I didn't get much time and I think it would have really helped me. (P6)

### Fieldnotes and reflexive accounts

A number of themes were identified from researcher fieldnotes and reflexive accounts, which were categorised into successes and challenges. Themes categorised as successes included benefits to researchers, benefits to women, and mutual benefits. Challenges included the timing of sessions, ongoing facilitation, funding and promoting the sessions.

#### Benefits to researchers

The women attending the sessions were from diverse cultural and social backgrounds—the range of insights and perspectives offered by the group exceeded perspectives from more ‘traditional’ methods of involvement. Some topics discussed at sessions led to conversations regarding cultural and religious practices, with women expressing a range of views and perspectives. This meant that researchers were able to explore diverse views on aspects of their research. Whilst the 20-min slot for involvement/engagement with public contributors limited potential for in-depth discussions, researchers were able to establish an initial relationship with the groups, which could be built upon with future involvement activities.

Although there were often new women attending the sessions, there was a core membership that attended regularly throughout their pregnancies. Stable membership meant that women formed relationships over time, which allowed for informal conversations and a relaxed and friendly atmosphere. Women also seemed to feel some ownership of the sessions and to take pride in welcoming researchers to sessions. As such, researchers were able to witness and take part in conversations that are not often encountered in formal health or research settings. For example, some women discussed negative attitudes towards health professionals and/or their care, some spoke about their complicated family relationships, and others expressed strong views on aspects of health in pregnancy (i.e. alcohol and cigarette use during pregnancy).

#### Benefits to women

Women expressed general enjoyment of Yoga for Bump sessions, and appreciated that classes were free: in the areas we held the sessions, costs for attending such activities were prohibitive for women with low incomes. The yoga element of the class was particularly popular.

Women attending Yoga for Bump sessions appeared to create a mutual peer support network, and reported having met socially outside of the group setting. Unlike some other maternity groups, women could attend the Yoga for Bump sessions at any stage of their pregnancy, and attendees reported the benefits of engaging with women at different gestations. Each week, attendees became increasingly open about their feelings, experiences, and fears, and discussions became longer and more detailed.

Having regular contact with a practising midwife from a local NHS Trust was also spoken about highly by participants during classes. Women voiced the benefits of informal contact with a health professional that they had built rapport with, and who subsequently knew a little about their medical history and lifestyle. Women also told facilitators of the benefits of hearing what other women were asking the midwife, and having the opportunity to learn about aspects of their care that they had not thought about previously Women also shared symptoms and experiences that they had not felt important or pressing enough to discuss with their own care team. In one instance, this led to referral to hospital for suspected deep vein thrombosis (DVT).

#### Mutual benefits

There was a good dynamic between the researcher, midwife and facilitator(s) during sessions, which appeared beneficial for both women’s knowledge, and the research engagement activities. Guest researchers were able to provide up-to-date evidence-based information on specific aspects of maternity research, which the midwife was then able to relate to guidance, other aspects of their maternity care, and how women might be able to benefit from research findings. For example, during a research engagement session on the benefits of vitamin use in pregnancy, the researcher provided the latest evidence-based advice, and the midwife advised women where they could get these vitamins cheaply or on prescription, and how to balance this vitamin intake with healthy eating. The question and answer session with the midwife then flowed naturally into a discussion of the benefits of healthy eating, nutrition, and exercise during pregnancy. The facilitator was able to navigate the relationship between researcher and midwife (who had not previously met), and also to facilitate the engagement element of the session. This gave women a holistic view of the research being presented, and provided researchers with an insight into the practical applications of their work.

#### Timing of sessions

The initial set-up of Yoga for Bump sessions was more difficult than anticipated as the midwife was only available to run classes on specific days/times and finding a location to accommodate this activity, especially within our budget, proved challenging. Based on discussions with our stakeholder group, which included a public contributor, we opted to hold an afternoon session and an early evening session.. However, after finding a suitable venue for the afternoon session (Hall Green), the only time that we were able to hold a class was 2:30–4 pm. It was quickly realised that this timing clashed with timings for collecting children from school, and therefore limited the potential for attendance by women who already had other children. This is reflected in the difference in the range and median for class sizes between the Perry Barr and Hall Green sessions, above. Although some women with other children did attend, this acted as a clear barrier, and we received a number of emails from women asking if other classes were available at this location. Due to limitations on funding and venue availability, we could not add further sessions. We were unable to change the timing of sessions as this would have been unfair to those already attending, and we were aware that our marketing of this time slot (on physical leaflets handed out by midwives and displayed in children’s centres/public spaces) could not be recalled.

#### Ongoing facilitation

Once established, Yoga for Bump sessions ran smoothly. There was a clear need, however, for the facilitator role. In addition to being responsible for logistical issues (opening-up, setting up the venue, bringing refreshments etc.) a key role for the facilitator was establishing and developing a relationship with pregnant women. This relationship-building activity was essential in developing trust, which is a critical element to public involvement. Researchers attending the sessions were able to capitalise on the already-established relationship between the facilitator and the group, and the facilitator played an important role in introducing the researcher and the research topic being presented. Additional facilitation included helping to convey concepts/terminology into more accessible language or encouraging women to ask questions or contribute to discussions.

The facilitator also played an important role in maintaining links between researchers and women attending Yoga for Bump sessions. In addition to ensuring women at the sessions were informed about the impact of their involvement, the facilitator was also able to provide updates on the progress of research projects and forward further comments/questions onto researchers.

Fulfilling the role of facilitator was very time-intensive. The study team worked physically on this project from 1:30 pm until 8 pm once a week, along with completing administration tasks (liaising with the midwife and venue, purchasing refreshments, promoting the sessions) on other days.

#### Funding

Initial set-up of the sessions included marketing and website costs (£350 total), payments for patient and public involvement activities (£280 total), as well as the purchase of 12 yoga mats (£120), and refreshments (£200). Costs per session included the salary for the yoga instructor/midwife (£100) and venue hire (£35–45). Facilitator time (approximately one day a week) was funded by NIHR CLAHRC WM, now recommissioned as NIHR ARC WM.

#### Promoting the sessions

Promotion of the Yoga for Bump sessions proved difficult with a limited budget, and attendance was initially low as a result. The study team created both Facebook and Twitter accounts for the sessions, however these only began to be used frequently towards the end of the project. Word of mouth was an effective mechanism for increasing attendance, and flyers for the sessions were handed out by some local midwives, although this was not consistent. As such, it took several months for the sessions to get embedded and promoted in the community, and attendance at the Hall Green session remained low. Earlier engagement with midwives may have facilitated better promotion.

## Discussion

This paper reflects on the development, implementation and initial evaluation of an innovative approach to engaging and involving pregnant and early postnatal women in maternity-related research. The initial evaluation suggests that Yoga for Bump sessions built collaborative and mutually beneficial research partnerships, providing a convenient and efficient way for researchers to involve and engage pregnant women in their research, with multiple benefits to both parties. This is an important finding, as it has previously been challenging to involve and engage pregnant women from diverse backgrounds in research [9].

In total, seven researchers attended Yoga for Bump sessions and had positive experiences of engaging and involving pregnant women. Of the researchers that attended the sessions, all reported that discussions with women at the sessions had added value to their work, and the majority reported changing an aspect of their research project as a result. The sessions also increased women’s overall awareness and interest in research, especially in the field of maternity care. This finding is reflected in previous work, where engagement activities have increased women’s curiosity in maternity-related research [29]. This is important, as active engagement with research can help dispel common myths around research participation and findings [30], and may encourage future involvement in research projects.

Women reported increasing their friendship and support networks, mixing with women at different stages of their pregnancy, feeling improvements in their health and wellbeing through the yoga element of the session, and having more knowledge about the evidence base behind their maternity care. Women also reported benefits from regular informal contact with a health professional that they had built rapport with, and who knew their history. This included the ability to ask questions about their pregnancies and care, as and when they arose: benefits which are not seen from traditional involvement activities.

Involving a midwife in research activities meant that women were able to discuss in further detail how the aims of the presented research related to their own experiences and care, and were consequently more likely to ask further questions. In a qualitative study on engaging pregnant women in observational research, authors found that a good working relationship with maternity care staff was seen to legitimise the presence of the researchers and instil trust in the pregnant women being recruited [31]. It is therefore possible that the engagement with, and implied ‘approval’ of the research topic by the midwife encouraged women’s engagement with the topic. This is important to acknowledge, as it highlights the importance of facilitator screening of researcher expressions of interest before attending sessions.

The community setting is an additional important feature of the Yoga for Bump approach. Due to budgetary and resource restrictions, research involvement activities have traditionally taken place in venues and at times most convenient to the research team rather than contributors. Activities held during the working day or at University/hospital may be prohibitive for pregnant women that work or have childcare commitments or for those that would need to travel. Longer involvement sessions may also prove challenging as pregnant women often have competing demands on their time. Formal structuring of involvement (i.e. meeting rooms, formal meeting procedures and minimal lay representation) has been suggested as exclusive or culturally imperialist, where the power dynamics inherent in social and health inequalities are repeated through models of involvement that prevent the creation of equal knowledge spaces [32, 33]. In our approach, an informal, local setting was adopted to enhance a feeling of mutual responsibility and to encourage women to lead discussions and ask questions. Rather than inviting women onto a formal setting such as a University campus, researchers attended community settings and adapted the timing and format to suit the needs of the women we aimed to engage (i.e. outside of the researchers’ normal working hours). This approach may be helpful when trying to achieve an equal relationship between members of the public and researchers [34]. Indeed, this approach enabled relationships to be built between the women, the midwife, and the facilitators. Women became increasingly interested in the research part of the sessions each week as their confidence grew, and these relationships strengthened. This appeared to result in more in-depth discussion around challenging and sensitive aspects of research.

A key challenge for researchers is to include a range of perspectives to shape research projects. The purposeful selection of different community settings for Yoga for Bump sessions enabled researchers to get insights and perspectives from pregnant women from diverse social and cultural backgrounds. Women attending Yoga for Bump sessions were younger and more ethnically diverse than the average public contributor: 50% of our attendees were non-white, compared to 16% of NIHR public contributors [15]. This is important, as increased involvement in research from diverse communities may facilitate increased diversity in research participation. This, in turn, will help ensure that evidence used to inform maternity care draws on data from all communities served, and may contribute towards addressing inequalities in pregnancy outcome. Women were able to provide unique insights and perspectives based on their personal experiences of pregnancy and accessing a range of maternity services. Researchers were able, therefore, to gain insights from a broad range of women, many of whom had no prior experience of involvement/engagement in research. Moreover, researchers were able to connect with women at different stages of gestation with very current experiences of pregnancy and accessing maternity services. This contrasts with much previous work, where pregnant women who engage with research are often those from higher educational and socioeconomic backgrounds, and are often less ethnically diverse than the general population [35, 36].

The inclusion of women with diverse experiences and perspectives, along with the collaborative and mutually beneficial research partnerships created by Yoga for Bump, is reminiscent of the ‘community engagement’ approach to research involvement and engagement put forward by the NIHR [34]. This approach emphasises the creation of alliances and relationships with communities, rather than just on an individual level. By creating a ‘community’ of pregnant women through Yoga for Bump sessions, we generated the potential for inclusive community engagement, where researchers could attend sessions to plan research in the community and with the community the research seeks to benefit. Despite the clear successes of the Yoga for Bump sessions, a number of challenges were experienced by the project team in terms of running these sessions. Acquiring a suitable venue and time slot for sessions with the available budget took longer than expected, and consequently one of the sessions took place during the time where some women were collecting other children from school. Due to challenges in finding a suitable venue and time, it took several months for the sessions to get embedded and promoted in each local community. As the project was only funded for one year, many of the women who attended towards the end of the project were disappointed that they were unable to continue these sessions throughout the remainder of their pregnancy.

In order to successfully implement a programme such as Yoga for Bump, careful consideration would be needed in terms of:

- The suitability of the venue and timing of the sessions
- The time and commitment of all those involved to make this a success (including appropriate facilitation and engagement from both a researcher and midwife, and administrative support)
- Adequate funding for the sessions to enable them to be embedded in communities
- Successful ways to promote knowledge of Yoga for Bump amongst all relevant groups:
  - women
  - local midwives, to promote the sessions
  - researchers, to promote attendance at sessions to share research

### Strengths and limitations

Yoga for Bump demonstrates an innovative approach to involving pregnant and early postnatal women with research; it is the first approach to combine an exercise class with healthcare advice and opportunities to involve and engage women with research, therefore mutually benefitting those involved. However, the use of an exercise class for these sessions also has limitations; it may be more likely to attract women who are motivated about their health, and may not be suitable for women with certain physical limitations. There may also have been a bias towards attendance by women with higher educational attainment, due to the choice of yoga as a prenatal exercise [37]. The inclusion of a local midwife may also have biased attendance towards women who value engagement with maternity services.

The funding for the pilot of this approach was only a year in duration, and as such only a small number of women and researchers took part. This, along with delays in Ethical approval, meant that the evaluation of this research was limited to questionnaire responses and fieldnotes. The reflexive fieldnotes were written by the project team funded to carry out the engagement work, and as such it is possible that sessions may have been viewed more positively than if they had been appraised by external researchers. In a future evaluation of this type of work, it would be useful to include individual interviews or focus groups with the women, stakeholders (including our PPI advisors) and researchers to gather richer data on their experiences.

Delays in Ethical approval for the evaluation of sessions also meant that we were only able to offer a survey to 20/67 of women who attended, rather than offering continuous opportunities for evaluation as originally planned. This may have introduced response bias, as all questionnaires were completed by women who had chosen to attend a session in December. To reduce the risk of bias, questionnaires were distributed by a member of ARC WM who had not been involved in the Yoga for Bump project.

This project was predominantly funded by the Wellcome Trust, and it is possible that this type of activity would not be feasible without such funding. The researchers taking part in the evaluation of these sessions suggested that they would consider paying to attend, and the costing of sufficient patient involvement is now a prerequisite to research funding by the NIHR and other grant organisations. However, this would be easier for established researchers with funded projects, but less so for researchers developing research ideas, or those carrying out research as part of a Masters or PhD programme of work.

## Conclusions

This paper presents an innovative approach to engaging pregnant and early postnatal women in research, which overcomes many of the existing barriers found in previous work. The research activity described demonstrates clear benefits to researchers and women alike. There is potential for this approach to be used to generate discussions around the important issues for women during their pregnancies and in the postnatal period, which could then be used to generate new research ideas. Further work is needed to design and evaluate similar approaches to engagement and explore potential funding avenues.

## Data Availability

The datasets used and/or analysed during the current study are available from the corresponding author on reasonable request.

## Abbreviations

ARC WM: Applied Research Collaboration, West Midlands
CLAHRC WM: Collaboration for Leadership in Applied Health Research and Care, West Midlands
DVT: Deep vein thrombosis
GP: General Practice
NHS: National Health Service
NIHR: National Institute for Health Research
UK: United Kingdom

## References

[1] Goodare H, Lockwood S (1999). Involving patients in clinical research. Improves the quality of research. BMJ.

[2] McAnuff J, Brooks R, Duff C, Quinn M, Marshall J, Kolehmainen N (2017). Improving participation outcomes and interventions in neurodisability: co-designing future research. Child Care Health Dev.

[3] Staniszewska S, Brett J, Mockford C, Barber R (2011). The GRIPP checklist: strengthening the quality of patient and public involvement reporting in research. Int J Technol Assess Health Care.

[4] Brett J, Staniszewska S, Mockford C, Herron-Marx S, Hughes J, Tysall C (2014). Mapping the impact of patient and public involvement on health and social care research: a systematic review. Health Expect.

[5] Rowe G, Frewer LJ (2000). Public participation methods: a framework for evaluation. Sci Technol Hum Values.

[6] National Institute for Health Research. Briefing notes for researchers - public involvement in NHS, health and social care research. 2021. https://www.nihr.ac.uk/documents/briefing-notes-for-researchers-public-involvement-in-nhs-health-and-social-care-research/27371#Definitions_of_involvement,_engagement_and_participation. Accessed 16 Aug 2021.

[7] Department of Health (2010). Equity and excellence: liberating the NHS.

[8] Partnership UPISD (2019). UK standards for public involvement: better public involvement for better health and social care research.

[9] Frew PM, Saint-Victor DS, Isaacs MB, Kim S, Swamy GK, Sheffield JS (2014). Recruitment and retention of pregnant women into clinical research trials: an overview of challenges, facilitators, and best practices. Clin Infect Dis.

[10] Morgan H, Thomson G, Crossland N, Dykes F, Hoddinott P (2016). Combining PPI with qualitative research to engage ‘harder-to-reach’populations: service user groups as co-applicants on a platform study for a trial. Res Involv Engagem.

[11] Bonevski B, Randell M, Paul C, Chapman K, Twyman L, Bryant J (2014). Reaching the hard-to-reach: a systematic review of strategies for improving health and medical research with socially disadvantaged groups. BMC Med Res Methodol.

[12] Smart A, Harrison E (2017). The under-representation of minority ethnic groups in UK medical research. Ethn Health.

[13] Beresford P. Beyond the usual suspects: Towards inclusive user involvement. Shaping our lives. 2013. https://www.shapingourlives.org.uk/documents/BTUSReport.pdf. Accessed 10 Jan 2021.

[14] Denegri S, Coldham T, Eglin S, Frost R, Kerridge L, Matthews R (2015). Going the extra mile: improving the nation’s health and wellbeing through public involvement in research.

[15] National Institute for Health Research. Taking stock–NIHR public involvement and engagement. 2019. https://www.nihr.ac.uk/documents/taking-stock-nihr-public-involvement-and-engagement/20566#NIHR_public_contributors%E2%80%99_feedback_survey. Accessed 23 July 2021.

[16] Draper E, Gallimore I, Smith L, Kurinczuk J, Smith P, Boby T (2019). MBRRACE-UK perinatal mortality surveillance report, UK perinatal deaths for births from January to December 2017.

[17] Knight M, Bunch K, Tuffnell D, Shakespeare J, Kotnis R, Kenyon S (2020). Saving lives, improving mothers’ care—lessons learned to inform maternity care from the UK and Ireland confidential enquiries into maternal deaths and morbidity 2016–18.

[18] Limb M (2021). Disparity in maternal deaths because of ethnicity is "unacceptable". BMJ.

[19] Boote J, Baird W, Beecroft C (2010). Public involvement at the design stage of primary health research: a narrative review of case examples. Health Policy.

[20] Staniszewska S, Jones N, Newburn M, Marshall S (2007). User involvement in the development of a research bid: barriers, enablers and impacts. Health Expect.

[21] Brett J, Staniszewska S, Mockford C, Seers K, Herron-Marx S, Bayliss H. The PIRICOM Study: a systematic review of the conceptualisation, measurement, impact and outcomes of patients and public involvement in health and social care research. 2010. https://www.ukcrc.org/wp-content/uploads/2014/03/Piricom+Review+Final+2010.pdf. Accessed 15 Jan 2021.

[22] Staley K (2009). Exploring impact: public involvement in NHS, public health and social care research. National Institute for Health Research.

[23] Oliver SR, Rees RW, Clarke-Jones L, Milne R, Oakley AR, Gabbay J (2008). A multidimensional conceptual framework for analysing public involvement in health services research. Health Expect.

[24] Harrison S, Alderdice F, Henderson J, Redshaw M, Quigley MA (2020). Trends in response rates and respondent characteristics in five National Maternity Surveys in England during 1995–2018. Arch Public Health.

[25] Kwon R, Kasper K, London S, Haas DM (2020). A systematic review: the effects of yoga on pregnancy. Eur J Obstet Gynecol Reprod Biol.

[26] Curtis K, Weinrib A, Katz J (2012). Systematic review of yoga for pregnant women: current status and future directions. Evid Based Complement Altern Med.

[27] Ministry of Housing, Communities & Local Government. National Statistics: English indices of deprivation 2015. 2015. https://www.gov.uk/government/statistics/english-indices-of-deprivation-2015. Accessed 23 July 2021.

[28] Ritchie J, Lewis J, Nicholls CM, Ormston R (2013). Qualitative research practice: a guide for social science students and researchers.

[29] Franck LS, McLemore MR, Williams S, Millar K, Gordon AY, Williams S (2020). Research priorities of women at risk for preterm birth: findings and a call to action. BMC Pregnancy Childbirth.

[30] Infanti JJ, O’Dea A, Gibson I, McGuire BE, Newell J, Glynn LG (2014). Reasons for participation and non-participation in a diabetes prevention trial among women with prior gestational diabetes mellitus (GDM). BMC Med Res Methodol.

[31] Muggli E, Curd H, Nagle C, Forster D, Halliday J (2018). Engaging pregnant women in observational research: a qualitative exploratory study. BMC Pregnancy Childbirth.

[32] Green G (2016). Power to the people: to what extent has public involvement in applied health research achieved this?. Res Involv Engagem.

[33] Locock L, Boylan A, Snow R, Staniszewska S (2017). The power of symbolic capital in patient and public involvement in health research. Health Expect.

[34] National Institute for Health Research. Building diverse and inclusive community research partnerships. 2020. https://rds-eoe.nihr.ac.uk/public-involvement/community-engagement/. Accessed 20 Aug 2021.

[35] Webster GM, Teschke K, Janssen PA (2012). Recruitment of healthy first-trimester pregnant women: lessons from the Chemicals, Health & Pregnancy study (CHirP). Matern Child Health J.

[36] Leung BM, McDonald SW, Kaplan BJ, Giesbrecht GF, Tough SC (2013). Comparison of sample characteristics in two pregnancy cohorts: community-based versus population-based recruitment methods. BMC Med Res Methodol.

[37] Cramer H, Frawley J, Steel A, Hall H, Adams J, Broom A (2015). Characteristic of women who practice yoga in different locations during pregnancy. BMJ Open.

